# Sedentary Conditions Promote Subregionally Specific Changes in Brain-Derived Neurotrophic Factor in the Rostral Ventrolateral Medulla

**DOI:** 10.3389/fphys.2021.756542

**Published:** 2021-10-13

**Authors:** Bozena E. Fyk-Kolodziej, Patrick J. Mueller

**Affiliations:** Department of Physiology, Wayne State University School of Medicine, Detroit, MI, United States

**Keywords:** brainstem, exercise, inactivity, BDNF, TrkB, p75NTR, rostral ventrolateral medulla

## Abstract

A sedentary lifestyle is the top preventable cause of death and accounts for substantial socioeconomic costs to society. The rostral ventrolateral medulla regulates blood pressure under normal and pathophysiological states, and demonstrates inactivity-related structural and functional neuroplasticity, which is subregionally specific. The purpose of this study was to examine pro- and mature forms of brain-derived neurotrophic factor (BDNF) and their respective receptors in the male rat rostral ventrolateral medulla (RVLM) and its rostral extension following sedentary vs. active (running wheels) conditions (10–12weeks). We used subregionally specific Western blotting to determine that the mature form of BDNF and its ratio to its pro-form were lower in more caudal subregions of the rostral ventrolateral medulla of sedentary rats but higher in the rostral extension when both were compared to active rats. The full-length form of the tropomyosin receptor kinase B receptor and the non-glycosylated form of the 75 kilodalton neurotrophin receptor were lower in sedentary compared to active rats. The rostrocaudal patterns of expression of the mature form of BDNF and the full-length form of the tropomyosin receptor kinase B receptor were remarkably similar to the subregionally specific patterns of enhanced dendritic branching, neuronal activity, and glutamate-mediated increases in sympathetic nerve activity observed in previous studies performed in sedentary rats. Our studies suggest signaling pathways related to BDNF within subregions of both the rostral ventrolateral medulla and its rostral extension contribute to cardiovascular disease and premature death related to a sedentary lifestyle.

## Introduction

Hypertension is a major public health concern in the U.S. and globally. Approximately 70 million adults in the United States have hypertension, which is also a significant risk factor for stroke, heart failure, and kidney failure (Kearney et al., 2005; Lloyd-Jones et al., 2009). Thus, the hypertensive condition results in high mortality but also major disabilities, loss of quality of life, and increased healthcare costs (Oparil et al., 2018; Bowling et al., 2019). Prompt diagnosis and effective treatments for hypertension are currently suboptimal, with ~30% of patients never achieving their target arterial pressure (Cutler et al., 2008). Therefore, there is clear need to understand the mechanisms by which hypertension develops, seek preventive measures to combat its progression, and to develop more optimal therapies once the chronic hypertensive state has been established.

Human hypertension is increasingly attributed to excessive activation of the sympathetic nervous system (Esler and Kaye, 2006; Fisher et al., 2009; Malpas, 2010). Under normal, healthy conditions sympathetic nerve activity is regulated by a region in the brainstem known as the rostral ventrolateral medulla (RVLM; Dampney, 1994; Guyenet, 2006; Schreihofer and Sved, 2011). The RVLM is also associated with sympathetic overactivity in several animal models of cardiovascular disease, including hypertension (Guyenet, 2006). Furthermore, an ever-increasing amount of evidence suggests that risk factors for hypertension, such as a lack of regular exercise, are associated with increased excitability of RVLM neurons (Martins-Pinge et al., 2005; Mischel et al., 2015; Subramanian and Mueller, 2016; Mueller et al., 2017). For example, RVLM neurons from sedentary vs. physically active rats demonstrate various forms of structural neuroplasticity, including increased dendritic branching (Mischel et al., 2014) and alterations in inhibitory and excitatory neurotransmitter receptors (Mueller et al., 2020; Fyk-Kolodziej et al., 2021), each suggestive of greater synaptic input and enhanced sympathoexcitatory and sympathoinhibitory responses following sedentary vs. physically active conditions. Interestingly, the pattern of increased dendritic branching in more rostral regions of the RVLM of sedentary rats corresponds *in vivo* to greater sympathoexcitatory responses to glutamate microinjections (Subramanian and Mueller, 2016) compared to active animals and greater neuronal activity in rostral regions of the RVLM in unexercised rats (Huereca et al., 2018). Therefore, mechanisms that enhance glutamatergic neurotransmission and structural neuroplasticity in a subregionally specific manner could serve as new therapeutic targets to attenuate inactivity-dependent neuroplasticity in the RVLM.

Brain-derived neurotrophic factor (BDNF) plays important roles in synaptic plasticity associated with learning and memory *via* upregulation of GluN receptors (Carvalho et al., 2008; Gomez-Pinilla et al., 2008) and enhancements in glutamatergic transmission (Lessmann et al., 1994; Lessmann, 1998; Lin et al., 1998; Sandoval et al., 2007). Recent work has also reported that BDNF signaling contributes to acute regulation of blood pressure and sympathetic outflow (Wang and Zhou, 2002; Clark et al., 2011; Wan et al., 2014; Erdos et al., 2015; Schaich et al., 2016, 2018). For example, overexpression of BDNF has been reported to augment; whereas, inhibition of BDNF signaling in the paraventricular nucleus of the hypothalamus (PVN) attenuates acute stress-induced increases in blood pressure (Erdos et al., 2015; Schaich et al., 2018).

Interestingly, the production of the mature form of BDNF (mBDNF) is dependent upon cleavage from its pro-form (proBDNF; Foltran and Diaz, 2016). The actions of proBDNF can oppose those of mBDNF, *via* its binding to the 75kDa neurotrophin receptor (p75NTR), which has been shown to reduce dendritic branching and contribute to pro-apoptotic pathways (Zagrebelsky et al., 2005; Yang et al., 2009, 2014). In addition, Sandoval et al. (2007) reported inhibitory effects of p75NTR vs. excitatory effects of the mBDNF receptor, TrkB, on NMDA receptor currents. Therefore, the ratio of mBDNF/proBDNF and their respective receptors may have important functional consequences in the overall synaptic plasticity occurring within a given brain region (Yang et al., 2009, 2014). However, we are unaware of any studies which have examined the collective expression of mBDNF, proBDNF, and their target receptors (TrkB and p75NTR) in the RVLM, particularly in the context of inactivity-related neuroplasticity. The lack of information regarding influences of inactivity on the RVLM is relevant given the stronger relationship between all-cause mortality and low cardiorespiratory fitness, when compared to other modifiable risk factors for cardiovascular disease, including smoking (Blair, 2009).

Importantly, unlike other brain regions such as the hippocampus (Caldeira et al., 2007; Kim et al., 2012; Vigers et al., 2012), the RVLM exhibits decreased excitatory neurotransmission (not increased) following periods of regular physical exercise when compared to sedentary conditions (Mueller, 2010; Mischel et al., 2015; Mueller et al., 2017). Although a recent study found no difference in BDNF in the RVLM of treadmill trained rats when examining the RVLM as a single structure (Lee et al., 2020), our recent work has emphasized a significant need to characterize subregional differences in RVLM neuroplasticity following sedentary vs. active conditions (Mischel et al., 2014; Subramanian and Mueller, 2016). As mentioned above, we have reported significant forms of neuroplasticity in phenotypically identified, presympathetic neurons of the RVLM. Several of these alterations occur uniquely in a region rostral to the caudal pole of the facial nucleus, which we have previously defined as the rostral extension of the RVLM (RVLM_RE_; Mueller et al., 2020; Fyk-Kolodziej et al., 2021). Therefore, the purpose of our study was to determine expression levels of proBDNF and mBDNF and receptors involved in their signaling pathways in different subregions of the RVLM and RVLM_RE_ of sedentary compared to physically active rats. Based on our previous reports of a subregional dependence of neuroplasticity in the RVLM (Mischel et al., 2014; Subramanian and Mueller, 2016; Mueller et al., 2020; Fyk-Kolodziej et al., 2021), we hypothesized that the expression of mBDNF, proBDNF, and their respective receptors (TrkB and p75NTR) would correspond to the subregional structural and functional neuroplasticity observed previously in sedentary vs. physically active rats.

## Materials and Methods

### Animal Models

Male Sprague–Dawley rats (*n*=26, Harlan/Invigo, Indianapolis, IN) were used in all experiments according to guidelines for animal care issued by the National Institute of Health and in accordance with an animal protocol approved by the Institutional Animal Care and Use Committee at Wayne State University (Animal Welfare Assurance Number A3310-01). Similar to our previous studies (Llewellyn-Smith and Mueller, 2013; Mischel et al., 2014; Mueller et al., 2020; Fyk-Kolodziej et al., 2021), rats were purchased at 4weeks of age (75–100g), and upon arrival were randomly divided into two experimental groups (*n*=13 each). Physically active rats were housed singly in standard cages and provided with in-cage access to running wheels 24h/day (Tecniplast, Eaton, PA, United States). Sedentary rats were housed singly in similar standard-sized cages without a running wheel. The running wheel activities of individual, physically active rats were recorded by bicycle computers attached to each cage (Sigma Sport, Olney, IL, United States). Running distances and durations were documented daily by laboratory personnel. As in our previous studies examining the effects of chronic sedentary vs. active conditions, rats were maintained under sedentary or active conditions for at least 10weeks but no more than 12weeks.

### Western Blotting

#### Microdissection and Tissue Preparation

The details of the RVLM tissue collection and preparation for Western blotting have been described in detail in our previous studies (Mueller et al., 2020; Fyk-Kolodziej et al., 2021). Briefly, a total of 26 rats from physically active and sedentary groups (*n*=13 each) were used for the entire study, with subgroups of animals used (*n*’s noted in figure legends) to assess expression of different proteins of interest. Animals were anesthetized deeply with a sub-lethal dose of Fatal-Plus (0.25ml/kg) and decapitated quickly. The whole brain was removed quickly from the scull, divided into the brainstem and forebrain using a rat brain matrix (Braintree Scientific, Braintree, MA, United States), and frozen on a stainless steel plate placed on dry ice. Brain segments were stored in a −80°C freezer until further cryosectioning.

**Figure 1:**
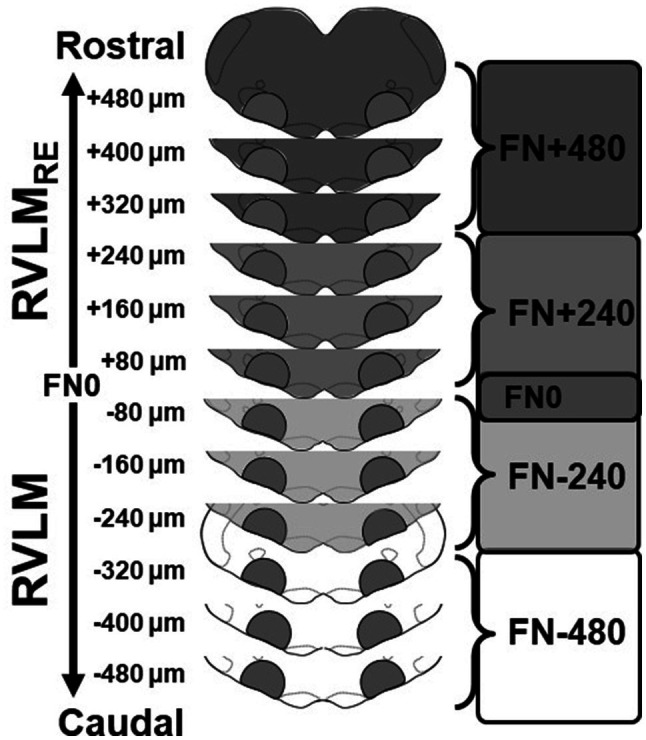
Schematic representation of sectioning, tissue punching, and pooling of bilateral samples of the rostral ventrolateral medulla (RVLM) and its rostral extension (RVLM_RE_). Medullae were cut into 80μm sections and punched bilaterally between the borders of the spinotrigeminal tract, nucleus ambiguus, and pyramids. Following cresyl violet staining of post-punched sections, punches were pooled according to their rostrocaudal position relative the caudal pole of the facial nucleus (designated FN0). Three punches were pooled to create two samples each containing approximately 240μm of the RVLM (FN-480 and FN-240; white and lighter gray, respectively) and the RVLM_RE_ (FN+240 and FN+480, medium and darker grey, respectively) again, related to their position relative to FN0.

Brainstem segments were cut in the coronal plane into 80μm sections using a cryostat (Microm HM 550; ThermoScientific, Waltham, MA, United States) and collected in serial order on uncoated, precleaned microscope slides. As shown in Figure 1, bilateral micropunches were retrieved along the rostrocaudal extent of the ventral medulla using a 17-gauge stainless steel blunt needle with a 1mm inner diameter. Bilateral micropunches were both placed in a single, chilled sample tube containing 20μl of ice-cold lysis buffer which consisted of 150mM NaCl, 1mM EDTA, 50mM Tris, 1% IGEPAL CA-630, and 0.1% Triton X-100, with the 1x Halt protease inhibitor cocktail (Pierce Biotechnology, Rockford, IL, United States).

Similar to our previous studies (Mueller et al., 2020; Fyk-Kolodziej et al., 2021), after tissue sections were punched, they were fixed on slides in 4% formaldehyde for 30min, washed three times with phosphate buffer, and stained with cresyl violet to visualize structural landmarks and verify the rostrocaudal boundaries of the RVLM and RVLM_RE_ regions. Using a standard rat atlas for reference (Paxinos and Watson, 2007), the section containing the caudal pole of the facial nucleus was identified (Bregma −12.12mm) and termed FN0, such that bilateral micropunches corresponding to each section could be designated serially into the more caudal RVLM or the more rostral RVLM_RE_ region. Two subregions of the RVLM were identified caudal to FN0 and were designated either FN-240 (containing the three bilateral 80μm punches from FN-80 to FN-240) or FN-480 (containing the three bilateral 80μm punches that is FN-320 to FN-480). Similarly, but rostral to FN0, punches designated for the RVLM_RE_ (three bilateral 80μm punches) were also pooled into two additional subregions: FN+240 and FN+480.

Pooled samples were placed on ice and subjected to sonication: twice for 3s each at 45% amplitude using an ultrasonic processor (Fisher Scientific, Waltham, MA, United States) with brief vortexing in between. In order to avoid overheating, cold water was used in the sonicator cuvette and water was changed between sonication of samples from each animal. After sonication, samples were centrifuged at 12,000rpm for 15min at 4°C and supernatants were collected in a fresh centrifuge tube for quantification of protein concentrations. Protein concentrations were determined using a bicinchoninic (BCA) protein assay kit (Pierce Biotechnology, Rockford, IL, United States) and measured at 562nm using Synergy H1 hybrid plate reader (Biotek, Winooski, VT, United States).

#### Immunoblotting

In all cases, protein samples from sedentary and physically active rats were loaded such that each gel contained samples from the four rostrocaudal levels (FN-480, FN-240, FN+240, and FN+480) obtained from one sedentary and one active rat. Depending on the protein of interest, 5–7.5μg samples of protein homogenate were separated on 12% (proBDNF and mBDNF), 7.5% (TrkB), or 10% (p75NTR) polyacrylamide gel using Tris/Glycine/SDS running buffer (Bio-Rad, Richmond, CA, United States) at 110V. After electrophoretic separation, proteins were transferred onto polyvinylidene difluoride (PVDF) membrane (Bio-Rad, Richmond, CA, United States) at 400mA for 2h in a cold room (proBDNF, mBDNF, and TrkB experiments) or at 15V overnight in a cold room (p75NTR experiments).

Similar to our previous studies (Mueller et al., 2020; Fyk-Kolodziej et al., 2021), the membranes were incubated in blocking solution containing 5% nonfat dry milk (Bio-Rad, Richmond, CA, United States), 3% normal goat serum (NGS) in 1xPBS, pH 7.4 with 0.1% Tween 20 (1xPBST) for 1h at room temperature. For TrkB experiments, 3% normal donkey serum (NDS) was used in the blocking solution instead of NGS.

After incubation in blocking solution, membranes were incubated overnight in one of the primary antibodies diluted in 1xPBS with 0.1–0.3% Tween 20, 2.5% dry milk, and 1% NGS or 1%NDS (TrkB experiments); rabbit anti-BDNF, rabbit anti-proBDNF, goat anti-TrkB, rabbit anti- p75NTR, or mouse anti-GAPDH. The details of each antiserum, such as source, employed immunogen, RRID number, and used dilution are provided in Table 1.

**Table 1:** Primary antibodies.

Antibody	Immunogen	Source, Cat.#, Species	RRID	Dilution
BDNF	Peptide (C)VLEKVPVSKQLK, corresponding to amino acids 166–178 of human BDNF	Alomone Labs, ANT-010, rabbit polyclonal	AB_2039756	1:200
proBDNF	Synthetic peptide from mouse proBDNF conjugated to immunogenic carrier protein	Invitrogen, OSB00016G, rabbit polyclonal	AB_10717050	1:500
TrkB	Recombinant mouse TrkB	Neuromics, GT15080, goat polyclonal	AB_2236287	1:1,500
p75NTR	Peptide CEEIPGRWITRSTPPE, corresponding to amino acids 188–203 of human p75NTR (extracellular domain)	Alomone Labs, ANT-007, rabbit polyclonal	AB_2039968	1:200
GAPDH Clone 6C5	GAPDH from rabbit muscle	EMD Millipore, MAB374, mouse monoclonal	AB_2107445	1:2,000

BDNF, brain-derived neurotrophic factor; GAPDH, glyceraldehyde 3-phosphate dehydrogenase; p75NTR, 75kDa neurotrophin receptors; proBDNF, pro form of BDNF; and TrkB, tropomyosin kinase B receptors.

After incubation in primary antibody, membranes were washed three times in 1xPBST (10min each), then were incubated for 1–2h at room temperature in the proper secondary antibody conjugated to horseradish peroxidase (HRP); (EMD Millipore, Temecula, CA, United States) diluted in 1xPBST with 2.5% dry milk and 1% NGS or 1%NDS (TrkB experiments). Table 2 contains details of secondary antibodies used in each set of experiments.

**Table 2:** Secondary antibodies.

Antibody	Immunogen	Source, Cat.#, Species	RRID	Dilution
anti-Rabbit, HRP conjugated	Whole rabbit IgG	Millipore, 12–348, goat polyclonal	AB_11214240	1:5,000
anti-Mouse, HRP conjugated	Whole mouse IgG	Millipore, 12–349, goat polyclonal	AB_390192	1:10,000
anti-Goat, HRP conjugated	Whole goat IgG	Millipore, AP180P, donkey polyclonal	AB_92573	1:10,000

HRP, horseradish peroxidase.

Protein bands were detected using enhanced chemiluminescent HRP substrate (ECL Western Blotting Detection Reagents, GE Healthcare, Piscataway, NJ, United States) and film autoradiography. Developed films were scanned and band densities were quantified using densitometric analysis and Image J software (NIH, Bethesda, Maryland). Immunoblot data are presented as a ratio of the protein of interest (mBDNF, proBDNF, TrkB, and p75NTR) to a loading control (GAPDH), the latter validated in our previous study for appropriate signal in the linear range of detection (Mueller et al., 2020; Fyk-Kolodziej et al., 2021).

#### Antibody Validation; Detection of Different Forms of BDNF, TrkB, and p75NTR

**Figure 2:**
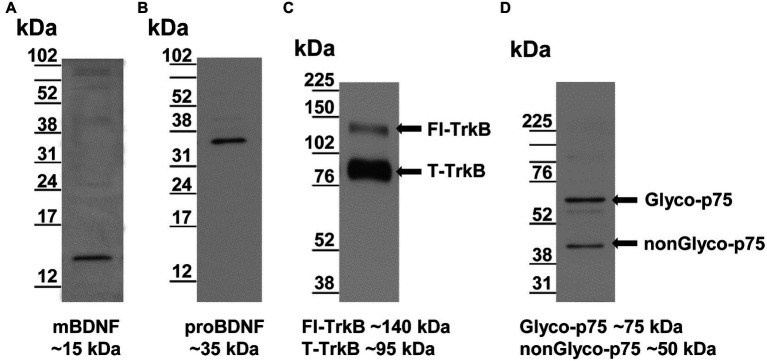
Validation of antibodies by Western blotting of RVLM samples. **(A)** The rabbit BDNF antibody recognizes a single band consistent with reported molecular weight ~15kDa (Seidah et al., 1996). **(B)** The rabbit proBDNF antibody recognizes a single band consistent with reported molecular weight of ~35kDa (Seidah et al., 1996). **(C)** The goat TrkB antibody recognizes two TrkB isoforms consistent with reported molecular weights of ~140 and ~95kDa for full length-form of TrkB (Fl-TrkB) and truncated form (T-TrkB), respectively (arrows; Klein et al., 1990; Escandon et al., 1994). **(D)** The rabbit p75 antibody representing the reported glycosylated (Glyco-p75, ~75kDa) and non-glycosylated (nonGlyco-p75, ~50kDa) forms, respectively (arrows; Grob et al., 1985; Kanning et al., 2003).

The rabbit BDNF antibody (RRID: AB_2039756) detects the mature form, mBDNF, as a single band at ~15kDa (Figure 2A), consistent with its reported molecular weight (Seidah et al., 1996). The rabbit BDNF antibody also detects a band ~150kDa, which very likely reflects mBDNF aggregates (not shown) and may occur during sample preparation. The rabbit proBDNF antibody (RRID: AB_10717050) detects only pro form of BDNF (proBDNF) as a single band at ~35kDa (Figure 2B), also consistent with its reported molecular weight (Seidah et al., 1996).

For detection of the two forms of BDNF (mBDNF and proBDNF), we used protein samples and membranes from the same sets of sedentary and physically active rats. First, the immunoblots were stained with a rabbit antibody that detects proBDNF form at ~35kDa (RRID: AB_10717050). The membranes were then washed several times in 1xPBS buffer and subsequently stained with a rabbit antibody (RRID: AB_2039756) that detects mBDNF at ~15kDa.

The goat TrkB antibody (RRID: AB_2236287) recognizes two TrkB isoforms, the full length form of the TrkB receptor (Fl-TrkB) and the truncated form (T-TrkB). The two bands detected by this antibody migrated to ~140 and ~95kDa (Figure 2C), consistent with the reported molecular weights for the Fl-TrkB and the T-TrkB isoforms, respectively (Klein et al., 1990; Escandon et al., 1994).

The rabbit p75NTR antibody (RRID: AB_2039968) raised against the extracellular domain recognizes two forms of the p75NTR receptor; that is, the glycosylated form (Glyco-p75) and the non-glycosylated form (nonGlyco-p75). This antibody detected bands at ~75 and ~50kDa (Figure 2D), which were consistent with the reported molecular weights for Glyco-p75 and nonGlyco-p75 forms, respectively (Grob et al., 1985; Kanning et al., 2003).

### Experimental Design and Statistical Analyses

The details of our experimental design were identical to those described in our most recent studies (Mueller et al., 2020; Fyk-Kolodziej et al., 2021). Briefly, Western blotting experiments were carried out in parallel in groups of age-matched sedentary and physically active rats. Similar to our previous studies, the number of animals used for each set of experiments was directed to maximize the amount of tissue used from each animal. We note in the Results section when tissue from the same animals was used for more than one experiment. For example, the immunoblotting experiments for the different forms of BDNF were each carried out on the same membranes containing samples from the same set of sedentary and physically active rats. Therefore, we analyzed the ratios of mBDNF/proBDNF, Fl-TrkB/T-TrkB, and Glyco-p75/nonGlyco-p75 independently to determine proportional differences in the varying forms of these proteins across the rostrocaudal extent of the RVLM and RVLM_RE_ regions.

Statistical analyses were performed using SigmaStat Version 3.5 software (Systat Software, San Jose, CA, United States). We used a two-way mixed ANOVA to compare body weights before and after sedentary or physically active conditions. The simple linear regression test was used to show a relationship between total running distance and protein expression within each subregion of the RVLM and RVLM_RE_ of physically active rats. Prior to running two-way mixed ANOVAs, all data were checked for normal distribution and passed Shapiro–Wilk normality test. In a few instances, noted in the Figure legends, data were log10 transformed in order to achieve normal distribution. We performed two-way mixed ANOVAs to compare between sedentary and physically active conditions (main effect of group, non-repeated measures) and subregional expression across the rostrocaudal extent of the ventrolateral medulla (main effect of rostrocaudal expression repeated measures). When significant interactions occurred between group and rostrocaudal expression, we performed multiple comparisons *post hoc* procedures (Holm–Sidak method) to determine differences between sedentary vs. active conditions within different rostrocaudal levels, and to determine differences between different rostrocaudal levels within two experimental groups. When significant main effects of rostrocaudal expression occurred in the absence of a significant interaction, we performed simple main effect testing to determine differences within the rostrocaudal distribution independent of group differences.

All data are presented as mean (SD) with *p*<0.05 indicating statistical significance. One data point collected in the T-TrkB dataset was deemed to be a mathematical outlier as it was two SDs from the mean and prevented the data set from achieving normal distribution and equal variance. To reduce its influence on the overall dataset and achieve normality and equal variance, we replaced the original value with the mean of the dataset with the original value included in the calculation. *p* values and degrees of freedom for each statistical test are reported in the Figure legends.

## Results

### Sedentary and Physically Active Animal Models

Similar to our previous studies (Subramanian et al., 2014; Subramanian and Mueller, 2016; Dombrowski and Mueller, 2017; Mueller et al., 2020; Fyk-Kolodziej et al., 2021), male Sprague–Dawley rats were maintained for 10–12weeks under active condition with in-cage running wheels (24h/day, *n*=13) or under sedentary condition where rats were housed in standard cages without a running wheel (*n*=13). In the first week of physical activity, rats were running ~3km per day for a total of ~84min per day. Over time, the average daily distance and daily duration gradually increased, reaching a peak after 5weeks, remaining at this steady-state for 3weeks, followed by a gradual decrease in distance and duration during the remaining weeks of activity (weeks 8–12). The average daily distances and daily durations during 10–12weeks of physical activity are included in Table 3.

**Table 3:** Running wheel activity.

Week of activity	Daily trip distance (km)	Daily duration (min)
1	2.75 (3.65)	84 (97)
2	3.43 (4.80)	95 (117)
3	3.66 (3.93)	98 (92)
4	4.05 (3.18)	103 (72)
5	4.76 (3.78)	116 (74)
6	4.17 (2.98)	111 (70)
7	4.14 (3.18)	107 (65)
8	3.61 (2.69)	92 (58)
9	3.25 (2.21)	85 (49)
10	2.54 (1.66)	72 (40)
11	2.24 (1.32)	65 (34)
12	2.18 (2.05)	66 (56)

Average daily running distance and average daily running duration per 1week over the time course of the study. Weeks 1–10 (*n*=13); weeks 11 and 12 (*n*=10). The smaller *n* values at weeks 11 and 12 indicate the removal of animals from the data set for fresh tissue harvesting at 10th week of the study. Values are expressed as means (SD). Km, kilometer; min, minute; SD, standard deviation.

The average total distance ran by physically active rats was 282 (216) km (*n*=13). As expected, and similar to our previous studies (Subramanian et al., 2014; Subramanian and Mueller, 2016; Dombrowski and Mueller, 2017; Mueller et al., 2020; Fyk-Kolodziej et al., 2021), sedentary rats had significantly higher body weights when compared with physically active animals at the end of the 10–12week study period [431 (24) vs. 376 (38) g, respectively]. Body weights were not different at baseline [95 (4) vs. 93 (7) g, respectively; *F* (1,24)=14.948, *p*<0.001 for interaction *via* two way mixed ANOVA; *p*<0.001 for *post hoc* test for ending body weight; *p*=0.792 for beginning body weights, *n*=13 per group].

### Expression of proBDNF and mBDNF Following Sedentary vs. Active Conditions

**Figure 3:**
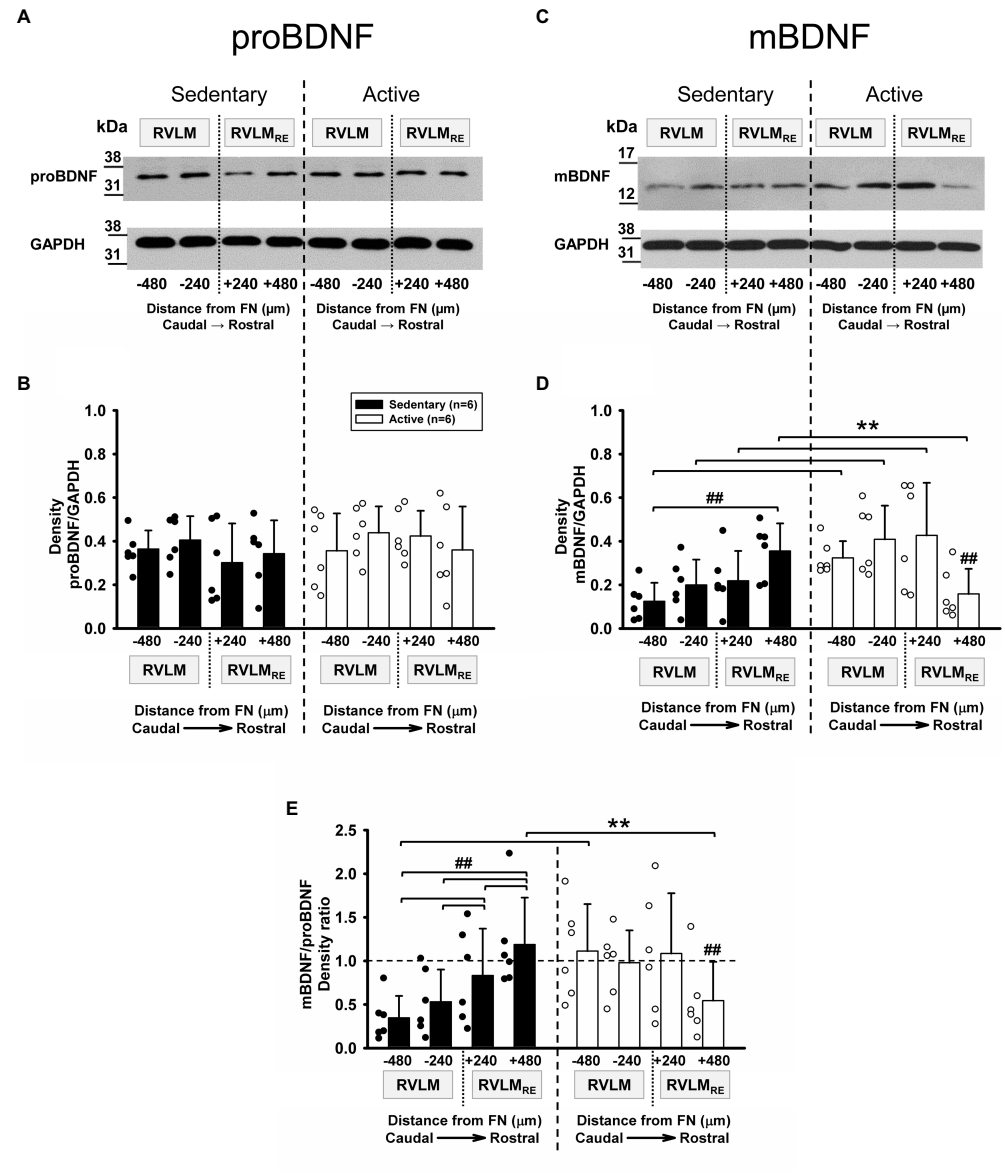
Pro-form (proBDNF) and mature form of BDNF (mBDNF) expression in the RVLM/RVLM_RE_ following 10–12weeks of sedentary vs. physically active conditions. **(A)** Representative Western blot of proBDNF and GAPDH expression at different rostrocaudal levels of the RVLM and the RVLM_RE_. **(B)** Group data from sedentary (black bars) vs. physically active (white bars) conditions (*n*=6 ea) demonstrate no significant difference in the expression of proBDNF in sedentary rats when compared to physically active animals in the RVLM/RVLM_RE_ [*F* (1,30)=0.31, *p*=0.59, main effect of group]. There was also no main effect of rostrocaudal distribution [*F* (3,30)=1.809, *p*=0.167] and the lack of significant interaction between experimental groups and rostrocaudal levels [*F* (3,30)=1.388, *p*=0.266] precluded further *post hoc* testing. **(C)** Representative Western blot of mBDNF and GAPDH expression at different rostrocaudal levels of the RVLM and the RVLM_RE_. **(D)** Group data from sedentary (black bars) vs. physically active (white bars) conditions (*n*=6 ea) show a significant interaction term between experimental groups and rostrocaudal levels [*F* (3,30)=11.182, *p*<0.001], and the further *post hoc* test for multiple factor comparisons (Holm–Sidak method), revealed that sedentary rats had significantly lower expression of mBDNF in both subregions of the RVLM (^**^p=0.021 for FN-480 and *p*=0.015 for FN-240) and in the FN+240 subregion of the RVLM_RE_ (^**^p=0.016). In contrast, the expression of mBDNF was significantly higher in sedentary animals in the most rostral FN+480 subregion of the RVLM_RE_ (^**^p=0.022). ##, significance for rostrocaudal comparisons within sedentary and active groups (see Supplementary Table S1 for *post hoc* results within experimental groups). **(E)** Group data showing significant interaction between main effects [*F* (3,30)=22.985, *p*<0.001] allowed for *post hoc* testing which revealed a significantly lower mBDNF/proBDNF ratio in sedentary rats in the most caudal FN-480 subregion of the RVLM (^**^p=0.005). In contrast, sedentary rats had a significantly higher mBDNF/proBDNF ratio in the most rostral subregion (FN+480) of the RVLM_RE_ (^**^p=0.024). ##, significance for rostrocaudal comparisons within sedentary and active groups (see Supplementary Table S1). Statistical results are from two-way mixed ANOVAs with significant differences between individual bars denoted with brackets and double symbols (^**^. ##) based on *post hoc* testing (see above). Data are presented as means (SD). Data in **(E)** were log10 transformed in order to achieve normal distribution prior to running two-way mixed ANOVAs.

Brain-derived neurotrophic factor is originally synthetized as the precursor proBDNF, which is cleaved to produce the mature form, mBDNF (for review, see Foltran and Diaz, 2016). In the present study, both proBDNF and mBDNF were expressed at each rostrocaudal level of the RVLM and RVLM_RE_ in both sedentary and physically active rats (Figures 3A,C, respectively). When compared to physically active animals, sedentary rats demonstrated no significant differences in the expression of proBDNF (*p*=0.59, main effect of group; Figure 3B). Neither the main effect of rostrocaudal distribution nor the interaction between group and rostrocaudal location reached significance (*p*=0.167 and *p*=0.266, respectively). Similar to proBDNF, there were no significant main effects of group or rostrocaudal distribution in mBDNF expression (*p*=0.119 and *p*=0.107, respectively). However, a significant interaction between group and rostrocaudal level (*p*<0.001) deferred our interpretation to *post hoc* testing. At individual rostrocaudal levels, mBDNF expression was significantly lower in sedentary vs. active rats in the FN-480 and FN-240 subregions of the RVLM (*p*=0.021 and 0.015, respectively), and in the FN+240 subregion of the RVLM_RE_ (*p*=0.016; Figure 3D). In contrast, in the most rostral subregion of the RVLM_RE_ (FN+480), mBDNF expression was significantly higher in sedentary vs. active rats (*p*=0.022; Figure 3D). Sedentary rats also demonstrated a distinguishable pattern of increasing expression from caudal to rostral levels as mBDNF expression was significantly higher at the most rostral subregion of the RVLM_RE_ (FN+480) compared to the most caudal subregion of the RVLM (FN-480, *p*=0.001). In contrast, mBDNF expression in active rats was characterized by the lowest expression in the most rostral subregion of the RVLM_RE_ (FN+480), particularly when compared to all regions located more caudally (Figure 3D, see Supplementary Table S1 for all *post hoc* results within sedentary and physically active groups).

Many studies have shown the opposing functional signaling of proBDNF and mBDNF forms (for review, see Zagrebelsky et al., 2020). While mBDNF has positive neurotrophic actions on neuron structure and number *via* Tropomyosin kinase B receptors (TrkB), proBDNF preferentially binds the 75kDa neurotrophin receptors (p75NTR) and results in effects opposite to those of mBDNF; that is, reduced neuroplasticity and apoptosis (Zagrebelsky et al., 2020). Since our immunoblotting experiments for proBDNF and mBDNF forms were carried out on the same membranes that contain samples from the same sets of sedentary and physically active rats, we reasoned that the balance between proBDNF and mBDNF would be indicative of the level of neuroplasticity in a given animal and differ significantly between active and sedentary animals.

Figure 3E represents mBDNF/proBDNF ratios from the same set of six sedentary rats compared to the same set of six physically active rats. There were no significant main effects of group or rostrocaudal location for the mBDNF/proBDNF ratio (*p*=0.349 and *p*=0.087, respectively). However, a significant interaction between groups and rostrocaudal levels (*p*<0.001) deferred us to *post hoc* testing. When comparing sedentary vs. physically active group, the *post hoc* test revealed a significantly lower mBDNF/proBDNF ratio in the most caudal subregion (FN-480, *p*=0.005) of the RVLM in sedentary rats but a significantly higher mBDNF/proBDNF ratio in the most rostral subregion (FN+480, *p*=0.024) of the RVLM_RE_ (Figure 3E) of sedentary rats. In addition, sedentary rats exhibited a significantly higher mBDNF/proBDNF ratio in the RVLM_RE_ subregions (FN+240 and FN+480) when compared to different subregions (FN-240 and FN-480) of the RVLM, i.e., [FN+240 vs. FN-240 (*p*=0.024); FN+240 vs. FN-480 (*p*<0.001) and FN+480 vs. FN-480; FN+480 vs. FN-240 (both *p*<0.001); and FN+240 vs. FN-480 and FN+240 vs. FN-240, *p*<0.001 and *p*=0.024, respectively]. Whereas the mBDNF/proBDNF ratio in physically active rats was significantly lower in the most rostral subregion (FN+480) of the RVLM_RE_ vs. all other subregions the RVLM and RVLM_RE_ (Figure 3E, see Supplementary Table S1 for all *post hoc* results within sedentary and physically active groups).

In the physically active group, the total running distance ranged from 113 to 312km over 10–12weeks of physical activity. In order to determine whether there was a correlation between the total running distance and protein expression levels, we performed linear regression analyses between total running distance for each rat and the corresponding protein expression values at each rostrocaudal level of the RVLM and the RVLM_RE_. Supplementary Table S4 summarizes a lack of significant correlations between the total running distance and expression levels of proBDNF, mBDNF, or the mBDNF/proBDNF ratio at each level of the RVLM or RVLM_RE_. These results suggest that the overall extent of physical activity as indicated by total running distance was not likely related to the level of proBDNF, mBDNF, or mBDNF/proBDNF ratio of expression in the RVLM or RVLM_RE_.

### Expression of T-TrkB and Fl-TrkB Following Sedentary vs. Active Conditions

**Figure 4:**
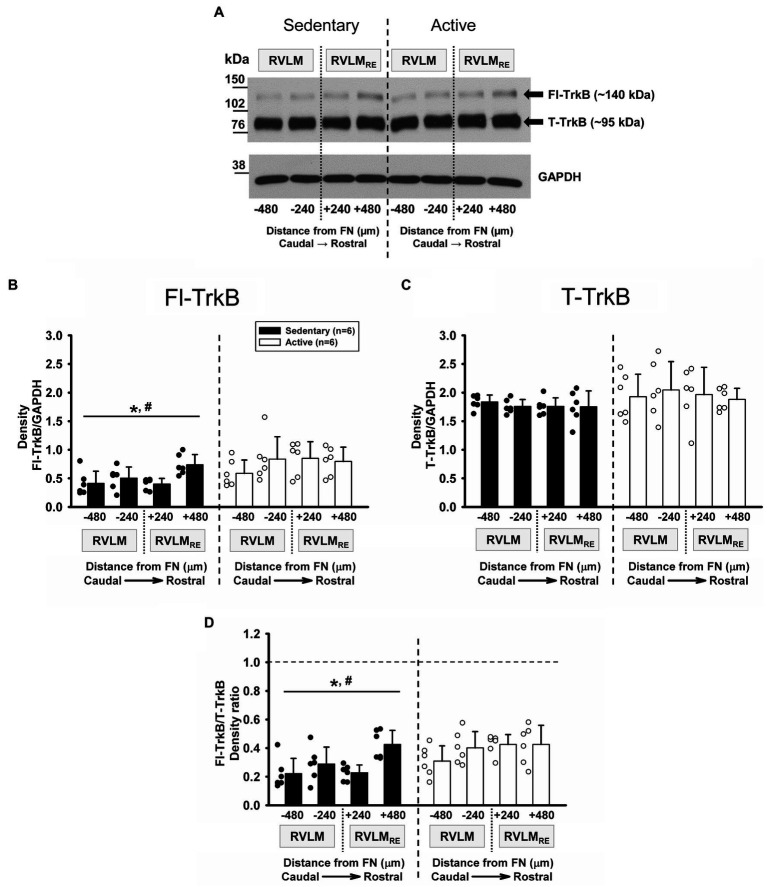
Expression of TrkB receptor isoforms in the RVLM/RVLM_RE_ following 10–12weeks of sedentary vs. physically active conditions. **(A)** Representative Western blot of Fl-TrkB (arrow ~140kDa) and T-TrkB (arrow ~95kDa) isoforms, and GAPDH expression (probed on same membrane) at different rostrocaudal levels of the RVLM and the RVLM_RE_. **(B)** Group data from sedentary (black bars) vs. physically active (white bars) conditions (*n*=6 ea) demonstrate an overall significantly lower expression of Fl-TrkB in sedentary compared to physically active animals [^*^, *F* (1, 30)=9.842, *p*=0.011, main effect of group]. There was also a main effect of rostrocaudal distribution [#, *F*(3,30)=3.967, *p*=0.017, see Supplementary Table S2 for all simple main effect comparisons]. The lack of significant interaction between experimental groups and rostrocaudal levels [*F* (3,30)=1.928, *p*=0.146] precluded further *post hoc* test testing. **(C)** Group data demonstrating no significant overall difference in the expression of T-TrkB in sedentary compared to active animals [*F* (1, 30)=1.345, *p*=0.273, main effect of group]. Neither a main effect of rostrocaudal distribution nor an interaction between groups and rostrocaudal levels reached significance [*F* (3,30)=0.425, *p*=0.737 and *F* (3,30)=0.601, *p*=0.619, respectively]. **(D)** Group data demonstrating a significantly lower Fl-TrkB/T-TrkB ratio in sedentary compared to active rats [^*^, *F* (1,30)=7.029, *p*=0.024, main effect]. A significant overall effect of rostrocaudal distribution on the Fl-TrkB/T-TrkB ratio suggested a lower ratio of expression in the caudal RVLM and higher expression in the rostral RVLM_RE_ [#, *F* (3,30)=6.080, *p*=0.002; main effect; see Supplementary Table S2 for rostrocaudal comparisons within collapsed groups]. The lack of significant interaction between main effects [*F* (3,30)=2.303, *p*=0.097] precluded further *post hoc* testing. Statistical results are from two-way mixed ANOVAs with lines and individual symbols (*. #) denoting significant main effects. Data in **(B)** were log10 transformed in order to achieve normal distribution prior to running two-way mixed ANOVAs.

Mature form of BDNF preferentially binds TrkB receptors which are alternatively spliced into two isoforms; the full-length form of TrkB (Fl-TrkB) and the truncated TrkB isoform (T-TrkB). The Fl-TrkB contains the intracellular tyrosine kinase domain and the extracellular domain, which is required for neurotrophin binding but the T-TrkB contains the same extracellular domain but is lacking the tyrosine kinase domain (Ohira and Hayashi, 2009). Both, the Fl-TrkB and the T-TrkB isoforms were expressed at each rostrocaudal level of the RVLM and the RVLM_RE_ following 10–12weeks of sedentary or physically active conditions with noticeably greater band intensity for T-TrkB compared to Fl-TrkB (Figure 4A). Group data from sedentary vs. physically active conditions (*n*=6 ea) demonstrate a significantly lower expression of Fl-TrkB in sedentary rats when compared to physically active animals in the RVLM/RVLM_RE_ (*p*=0.011, main effect of group; Figure 4B). In addition, there was significant main effect of rostrocaudal distribution (*p*=0.017; Figure 4B). The simple main effect testing (Holm-Sidak method) showed significantly lower Fl-TrkB expression level in the most caudal subregion (FN-480) of the RVLM vs. the most rostral subregion (FN+480) of the RVLM_RE_ independent of experimental groups (*p*=0.002; Figure 4B, see Supplementary Table S2 for all simple rostrocaudal main effect comparisons). Lastly, the interaction term between experimental groups and rostrocaudal levels did not reach significance (*p*=0.146), which precluded *post hoc* testing.

In contrast to Fl-TrkB expression, T-TrkB was not significantly different between sedentary vs. physically active conditions (*p*=0.273, main effect of group; Figure 4C) and there was no main effect of rostrocaudal distribution (*p*=0.737). In addition, there was no significant interaction term between experimental groups and rostrocaudal levels (*p*=0.619), which precluded *post hoc* testing.

Since Fl-TrkB and T-TrkB isoforms contain the extracellular neurotrophin binding domain and therefore the mature BDNF can bind both isoforms, the ratio of Fl-TrkB and T-TrkB may be indicative of the overall action mBDNF *via* TrkB receptors. As mentioned above, there was noticeably greater band intensity for T-TrkB compared to Fl-TrkB in both sedentary and active rats suggesting a higher expression of T-TrkB. In addition, sedentary rats showed a significantly lower Fl-TrkB/T-TrkB expression ratio compared to physically active rats (*p*=0.024, main effect of group; Figure 4D). There was also a significant main effect of rostrocaudal distribution for the Fl-TrkB/T-TrkB ratio (*p*=0.002; Figure 4D) and simple main effect testing revealed significantly higher Fl-TrkB/T-TrkB expression ratio in the most rostral subregion (FN+480) of the RVLM_RE_ vs. the most caudal subregion (FN-480) of the RVLM independent of experimental groups (*p*<0.001; Figure 4D; see Supplementary Table S2 for all simple rostrocaudal main effect comparisons). The interaction term for group and rostrocaudal level did not reach significance (*p*=0.097), which precluded further *post hoc* testing.

Similar to mBDNF expression and the mBDNF/proBDNF expression ratio observed in sedentary rats, Fl-TrkB expression and the Fl-TrkB/T-TrkB expression ratio in sedentary rats were also significantly higher in the most rostral subregion of the RVLM_RE_ (FN+480) compared to the most caudal subregion (FN-480) of the RVLM (compare Figures 4B,D with Figures 3D,E, respectively).

In the physically active group, the total running distance ranged from 113 to 591km over 10–12weeks of physical activity. Overall, there were no significant correlations between the Fl-TrkB, T-TrkB, and Fl-TrkB/T-TrkB protein expression levels and total running distance in most of the rostrocaudal levels of the RVLM and the RVLM_RE_ (Supplementary Table S4). However, in the FN-240 subregion of the RVLM, the Fl-TrkB expression and the Fl-TrkB/T-TrkB ratio showed significant positive correlation with the total running distance (*p*=0.033 and *p*=0.011, respectively). These results suggest that the amount of physical activity could contribute in some degree to the expression of Fl-TrkB and Fl-TrkB/T-TrkB levels in the RVLM.

### Expression of Glyco-p75 and nonGlyco-p75 Receptors Following Sedentary vs. Active Conditions

**Figure 5:**
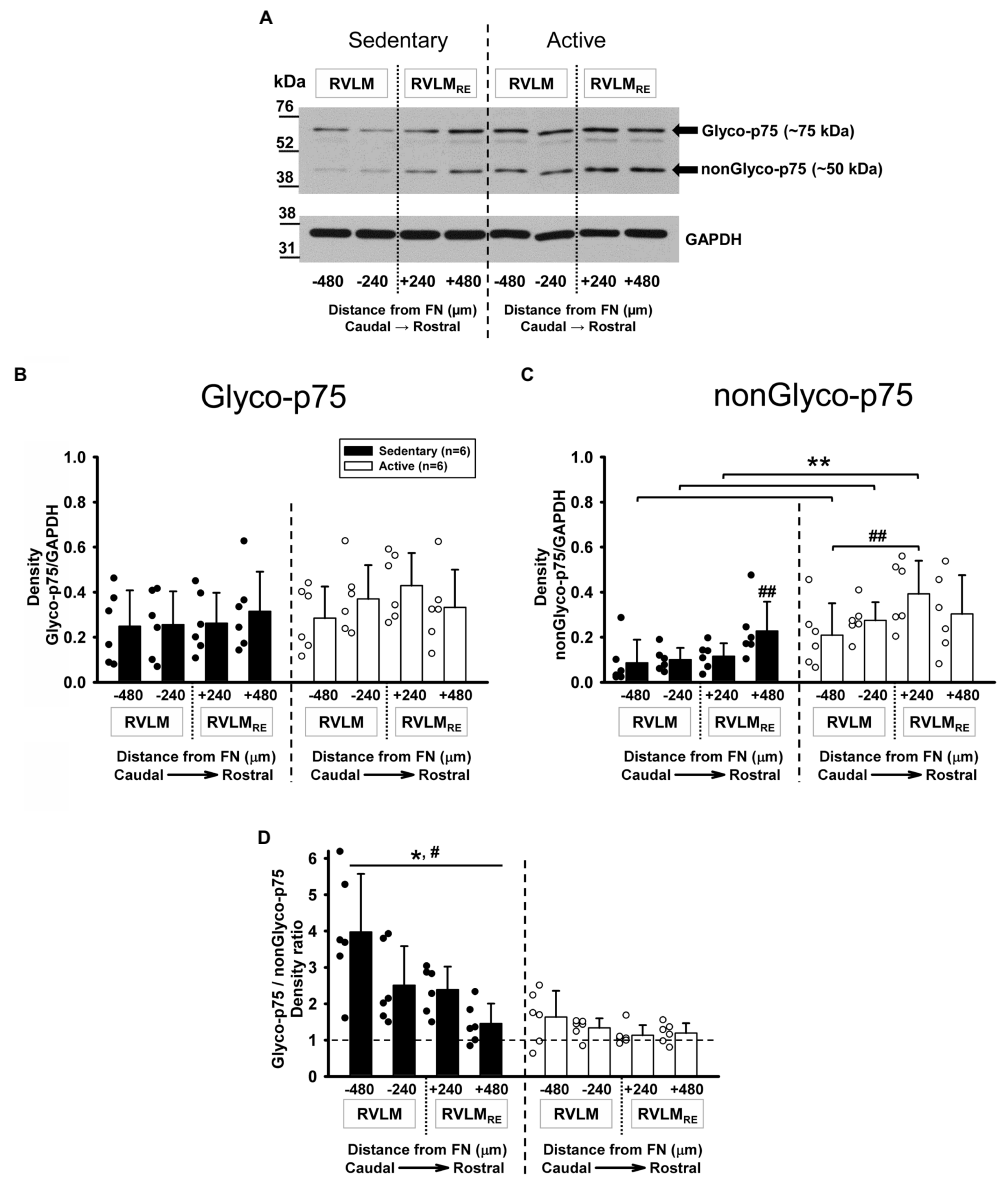
Expression of Glycosylated (Glyco-p75) and non-Glycosylated (nonGlyco-p75) forms of the p75NTR receptor in the RVLM/RVLM_RE_ following 12weeks of sedentary vs. physically active conditions. **(A)** Representative Western blot of Glyco-p75 (arrow ~75kDa), nonGlyco-p75 (arrow ~50kDa), and GAPDH expression at different rostrocaudal levels of the RVLM and the RVLM_RE_. **(B)** Group data from sedentary (black bars) vs. physically active (white bars) conditions (*n*=6 ea) demonstrate no significant overall difference in the expression of Glyco-p75 in sedentary compared to active animals [*F* (1, 30)=1.150, *p*=0.309, main effect of group] and there was also no overall significant effect of rostrocaudal distribution [*F* (3,30)=1.877, *p*=0.155; main effect]. The interaction between experimental groups and rostrocaudal levels did not reach a significance [*F* (3,30)=2.029, *p*=0.131], which precluded further *post hoc* testing. **(C)** Group data from sedentary vs. physically active conditions demonstrate a significant interaction term [*F* (3,30)=3.384, *p*=0.031] and revealed a significantly lower expression of nonGlyco-p75 in both RVLM subregions (^**^p=0.004 for FN-480 and *p*=0.006 for FN-240) and in the FN+240 subregion of the RVLM_RE_ (^**^, *p*=0.001) of sedentary rats. Sedentary rats showed significantly higher expression of nonGlyco-p75 in the FN+480 of the RVLM_RE_ compared to the FN+240 (##, *p*=0.012) and both RVLM subregions (##, *p*=0.004 for FN-240 and *p*<0.001 for FN-480). Physically active rats exhibited significantly higher expression of nonGlyco-p75 in the FN+240 subregion of RVLM_RE_ compared with the FN-480 of RVLM (##, *p*=0.006). See Supplementary Table S3 for results of all rostrocaudal comparisons within groups. **(D)** Group data showing that the Glyco-p75/nonGlyco-p75 ratio was overall significantly higher in sedentary rats vs. physically active [*, *F* (1,30)=20.829, *p*=0.001, main effect] and that there was an overall significant main effect of rostrocaudal distribution [#, *F* (3,30)=7.205, *p*<0.001, main effect]. Simple main effect testing revealed a significantly higher Glyco-p75/nonGlyco-p75 ratio in the most caudal FN-480 subregion of the RVLM compared with two subregions (FN+240 and FN+480) of the RVLM_RE_ (*p*=0.009 and *p*<0.001, respectively, see Supplementary Table S3 for all simple main effect comparisons). The interaction between main effects did not reach a significance [*F* (3,30)=2.895, *p*=0.051] which precluded further *post hoc* testing. Data in **(C,D)** were log10 transformed in order to achieve normal distribution prior to running two-way mixed ANOVAs.

Although proBDNF can be cleaved intra- or extracellularly to release mBDNF (Foltran and Diaz, 2016), proBDNF is also secreted and binds preferentially to the p75NTR receptors, where it regulates neuron structure, function, and number in an inhibitory manner (Yang et al., 2009). It is also well known, that glycosylation plays an important role in p75NTR receptor function since O-glycosylation is required for transport to the plasma membrane (Yeaman et al., 1997). Glyco- and nonGlyco-p75 receptors were present at each rostrocaudal level of the RVLM and the RVLM_RE_ following 12weeks of sedentary or physically active conditions (Figure 5A). There was no significant difference overall in the expression of Glyco-p75 in sedentary rats when compared to physically active animals in the RVLM/RVLM_RE_ (*p*=0.309, main effect of group; Figure 5B). In addition, neither the main effect of rostrocaudal location nor the interaction between experimental groups and rostrocaudal levels reached significance (*p*=0.155 and *p*=0.131, respectively; Figure 5B).

In contrast to Glyco-p75, the nonGlyco-p75 form was significantly lower in the RVLM/RVLM_RE_ of sedentary rats when compared to physically active animals (*p*=0.008, main effect of group) and there was a significant main effect of rostrocaudal distribution (*p*<0.001). The significant interaction between main effects (*p*=0.031) deferred us to further *post hoc* testing for multiple factor comparisons. Sedentary rats had a significantly lower expression of nonGlyco-p75 in both RVLM subregions (*p*=0.004 for FN-480 and *p*=0.006 for FN-240) and in the FN+240 subregion of the RVLM_RE_ compared to active rats (*p*=0.001, Figure 5C).

In addition, sedentary rats showed a significantly higher expression of nonGlyco-p75 in the most rostral subregion (FN+480) of the RVLM_RE_ compared to all other subregions of the RVLM/RVLM_RE_. In the physically active rats, nonGlyco-p75 was significantly higher at FN+240 of the RVLM_RE_ compared to the FN-480 of the RVLM (*p*=0.006; Figure 5C; see Supplementary Table S3 for all *post hoc* results within sedentary and physically active groups).

The ratio of Glyco-p75/nonGlyco-p75 expression was significantly higher in the sedentary vs. physically active rats (*p*=0.001, main effect; Figure 5D). There was also a significant main effect of rostrocaudal distribution (*p*<0.001; Figure 5D). Both sedentary and physically active rats exhibited a characteristic decreasing expression pattern of Glyco-p75/nonGlyo-p75 from caudal to rostral regions, which was more apparent in sedentary rats. Simple main effect testing (Holm–Sidak method) revealed a significantly higher expression of Glyco-p75/nonGlyco-p75 in the most caudal subregion (FN-480) of the RVLM vs. two rostral subregions (FN+240 and FN+480) of the RVLM_RE_ in both experimental groups (Figure 5D, see Supplementary Table S3 for all simple main effect comparisons). An interaction between groups and rostrocaudal levels did not reach a significance (*p*=0.051), precluding further *post hoc* testing.

In sedentary rats, Glyco-p75/nonGlyco-p75 expression levels above 1.00 at each rostrocaudal subregion of the RVLM/RVLM_RE_ suggest a higher expression of the glycosylated form of p75 compared to non-glycosylated form, but in physically active rats, the noticeable Glyco-p75/nonGlyco-p75 expressions near to 1.00 at each rostrocaudal subregion of the RVLM/RVLM_RE_ may indicate more balanced expression levels of Glyco- and nonGlyco-p75 forms (Figure 5D).

In the physically active group tested for Glyco-p75, nonGlyco-p75, and Glyco-p75/nonGlyco-p75 expression levels, the total running distance ranged from 77 to 680km over 12weeks of physical activity. Neither the Glyco-p75, nonGlyco-p75, nor Glyco-p75/nonGlyco-p75 expression levels in any RVLM or RVLM_RE_ subregions displayed the significant correlations with total running distance (Supplementary Table S4). These results suggest that the degree of physical activity did not contribute to the p75 expression level in the RVLM/RVLM_RE_.

## Discussion

The purpose of our study was to determine expression levels of proteins involved in proBDNF and mBDNF signaling pathways in different subregions of the RVLM/RVLM_RE_ of sedentary compared to physically active rats. The most important findings of our study are: (1) proBDNF and mBDNF, and the different forms of their respective receptors are expressed throughout different subregions of the RVLM, including its rostral extension, termed here and in our previous studies as the RVLM_RE_ (Mueller et al., 2020; Fyk-Kolodziej et al., 2021). (2) The levels and patterns of expression of proBDNF, mBDNF, and their respective receptors were dependent on both subregional location and whether the animals had performed voluntary physical exercise or remained sedentary for 10–12weeks. (3) Specifically, mBDNF and the ratio of mBDNF/proBDNF were significantly lower in the more caudal subregions of sedentary animals but significantly higher in the more rostral region of the RVLM_RE_ when compared to active rats. (4) In contrast, expression levels of proBDNF were not significantly different between sedentary compared to active rats but the full-length form of the Trk-B receptor (Fl-TrkB) and the non-glycosylated form of the p75NTR receptor (nonGlyco-p75) were significantly lower in sedentary animals. Fl-TrkB and nonGlyco-p75 were also expressed in a characteristic caudal to rostral expression pattern. The increasing expression pattern of mBDNF and the Fl-TrkB receptor from caudal to rostral regions is remarkably consistent with the pattern of increased dendritic branching and increasing in neuronal activity observed previously in sedentary rats (Mischel et al., 2014; Huereca et al., 2018). It is also uniquely consistent with enhanced glutamate-mediated increases in splanchnic sympathetic nerve activity, which have been reported to occur in more rostral vs. caudal regions of the RVLM in sedentary vs. active rats (Subramanian and Mueller, 2016). As discussed in detail below, for the first time, we report subregional expression of mBDNF and its receptor related to physical inactivity-dependent neuroplasticity in regions containing presympathetic neurons involved in sympathetic nerve activity and blood pressure regulation. Based on these findings and others, we hypothesize that the BDNF signaling pathway within the RVLM may be a viable target for the treatment of hypertension and sympathoexcitation occurring in sedentary individuals.

Our observation that mBDNF and the TrkB receptor are expressed in the RVLM is highly consistent with previous studies. Both BDNF mRNA and protein have been reported in the RVLM of rats using different detection techniques including RT-PCR, Western blotting, and immunohistochemistry (Wang and Zhou, 2002; Chan et al., 2010; Wang et al., 2019; Lee et al., 2020). Similarly, both the full-length and truncated forms of the TrkB receptor have been identified *via* Western blotting techniques in the RVLM (Wang et al., 2019; Lee et al., 2020). To the best of our knowledge, we are the first laboratory to quantify the relative level of protein expression of pro-BDNF and its complementary p75 receptor, both in its glycosylated and non-glycosylated form, in the ventrolateral medulla. Because of the opposing actions of mBDNF vs. proBDNF on neuroplasticity *via* TrkB and p75NTR receptors, respectively (Yang et al., 2009, 2014), the overall level and pattern of expression mBDNF vs. proBDNF and their respective receptors would be expected to directly influence the direction, magnitude and type of neuroplasticity occurring in a given brain region or subregion (discussed below).

Our finding that mBDNF expression was dependent on sedentary vs. active conditions and subregional location in the RVLM/RVLM_RE_ is unique based on findings in the current literature. Previous studies have reported an increase in levels of mBDNF following chronic exercise in other brain regions, particularly in the hippocampus, where it has been associated with improvements in memory and cognition (Adlard et al., 2004; Caldeira et al., 2007; Kim et al., 2012; Vigers et al., 2012). In contrast, Lee et al. (2020) found no differences in mBDNF levels in the RVLM of rats which had performed 4weeks of treadmill exercise following induction of myocardial infarction (Lee et al., 2020). It is possible that expression of mBDNF in the RVLM is not sensitive to chronic exercise under conditions of myocardial infarction. However, mBDNF expression in the RVLM does appear to be affected by more acute perturbations, considering direct injections of angiotensin II into the RVLM increases BDNF mRNA (~0.5-fold) and protein (~2–3-fold) levels within 4h of administration (Chan et al., 2010). In the present study, mBDNF was higher in active animals in more caudal regions but lower in the more rostral region of the ventrolateral medulla. Subregionally dependent differences in expression of mBDNF observed in this study may explain why Lee et al. (2020) found no significant differences in the overall levels of mBDNF levels in post-myocardial infarction rats, particularly when the RVLM was examined as an entire region. Other differences in animal models, strains of rat and type, and duration of exercise protocols could influence the outcomes of each study as well. Future studies could consider rostrocaudal differences in the expression of mBDNF following post-myocardial infarction to resolve this potential discrepancy.

The expression pattern of mBDNF is remarkably consistent with many aspects of our previous studies, which have examined neuroplasticity in the RVLM of sedentary vs. physically active animals. In 2014, we reported that bulbospinal C1 neurons, known for their role in control of sympathetic outflow (Schreihofer and Sved, 2011; Guyenet et al., 2013), had significantly greater dendritic branching, dendritic length, and dendritic number in sedentary compared to physically active rats (Mischel et al., 2014). In that same study, we reported that dendritic branching was subregionally selective; that is, sedentary rats showed significantly greater dendritic branching compared to active rats but only in the more rostral subregions of the RVLM. Based on consistent anatomical and biochemical differences in the region rostral to the caudal pole of the facial nucleus, we have distinguished this area as the RVLM_RE_ here and in more recent studies (Mueller et al., 2020; Fyk-Kolodziej et al., 2021). Furthermore, consistent with anatomical and biochemical evidence, we have also reported that sedentary conditions enhance sympathoexcitatory responses to glutamatergic activation of RVLM neurons *in vivo*, specifically in more rostral regions when compared to active animals (Subramanian and Mueller, 2016). Interestingly, mBDNF has been proposed to augment glutamatergic transmission in other brain regions (Lessmann et al., 1994; Lessmann, 1998; Lin et al., 1998; Suen et al., 1998; Ding et al., 2006; Gomez-Pinilla et al., 2008). However, a direct mechanistic link between increased glutamatergic neurotransmission and increases in BDNF expression in the RVLM has not yet been made. In our recent study, we reported increased levels of expression of NMDA receptor subunits GluN1 and GluN2B in the RVLM of sedentary compared to physically active rats (Fyk-Kolodziej et al., 2021). In particular, significantly higher expression of GluN2B subunits in sedentary animals was restricted to the most rostral subregions of RVLM_RE_. Thus, it is very reasonable to suspect that the coincident increased dendritic branching (Mischel et al., 2014), enhanced sympathoexcitatory responses (Subramanian and Mueller, 2016), and increased NMDA receptor expression levels (Fyk-Kolodziej et al., 2021) in the rostral portions of the ventrolateral medulla are linked mechanistically to increased expression levels of mBDNF and Fl-TrkB in sedentary animals observed in the present study.

To our knowledge, this is the first study to report proBDNF expression in the RVLM of sedentary and active rats, which provides several insights into mechanisms of (in)activity-related neuroplasticity. First, the production of proBDNF is a necessary step in the formation of mBDNF (Foltran and Diaz, 2016). Second, proBDNF can oppose the actions of mBDNF, *via* activation of the p75NTR receptor to reduce dendritic branching and contribute to pro-apoptotic pathways (Yang et al., 2009, 2014). Third, O-glycosylation of p75NTR receptors is required for transport to the plasma membrane based on *in vitro* studies (Yeaman et al., 1997). Therefore, given the overall similar expression of proBDNF but higher expression of non-Glyco-p75 receptors in the rostral RVLM_RE_ subregion compared to the more caudal RVLM subregion (Figure 5C, ##) in sedentary rats, the simplest interpretation is that there may be reduced proBDNF-mediated signaling to inhibit neuroplasticity in the RVLM_RE_ subregion. Furthermore, as proBDNF expression was more consistent across the rostrocaudal extent and mBDNF expression increased in a caudal to rostral pattern, a similar pattern of an increased mBDNF/proBDNF ratio is present from the caudal RVLM to the more rostral RVLM_RE_ and specifically in sedentary animals (Figure 3E). In contrast, the mBDNF/proBDNF ratio is fairly consistent across the caudal to rostral axis in active animals, but is significantly lower in the most rostral RVLM_RE_, again the region that appears to have the greatest differences in the increased dendritic branching (Mischel et al., 2014), glutamate-mediated sympathoexcitation (Subramanian and Mueller, 2016), and NMDA receptor subunit expression in sedentary compared to physically activity animals (Fyk-Kolodziej et al., 2021). Collectively, the increasing ratio of mBDNF/proBDNF in sedentary rats suggests mechanisms more favorable toward increases in excitatory neuroplasticity (Yang et al., 2009, 2014).

We observed no significant correlation between running distances and the expression of proBDNF, mBDNF, and most of the different forms of their respective receptors in the current study. Previous studies have demonstrated disparate findings in whether BDNF expression level is related to the amount of exercise performed. For example, in a study similar to the present one, Oliff et al. (1998) reported that the distance run by rats provided access to running wheels was significantly correlated with hippocampal mRNA expression of BDNF. In contrast, Groves-Chapman et al. (2011) reported more recently that there was no relationship between BDNF mRNA expression and running distance in the hippocampus of rats provided a running wheel for 3weeks. In the present study, we only observe significant positive correlations between running distances and both Fl-TrkB expression and the Fl-TrkB/T-TrkB ratio. Differences in brain regions, assessing mRNA vs. protein expression and duration of physical activity are only a few factors to consider comparing across studies (Oliff et al., 1998; Groves-Chapman et al., 2011). For example, the average daily durations were higher in the present study compared to two of our previous studies and is reflective of using a voluntary model of exercise vs. a forced exercise model or a fixed duration exercise model or both. In addition, some of our older publications (Mischel and Mueller, 2011) report similar results to the current study. Nonetheless, there is a clear need for more studies to examine these relationships and the impact they produce on neuronal structure and function.

In summary, we have identified intriguing subregional patterns of neurotrophin expression in the RVLM/RVLM_RE_ of sedentary and physically active animals that are consistent with a mechanistic link to previous studies reporting structural and functional neuroplasticity in sedentary vs. physically active conditions. The experimental limitations of Western blotting techniques, although highly rigorous and consistent, do restrict our interpretations to some degree. For example, although it is well established that neurons produce strong BDNF-mediated signals through Fl-TrkB receptors, there are publications describing the role of TrKB/BDNF signaling in non-neuronal cells, such as astrocytes (Saba et al., 2018) and glia (Rose et al., 2003). Therefore, further studies will be required to test our hypotheses more directly including the use of cell-type specific immunohistochemistry; assessments of the levels and types of post-translational modifications; and proteolytic processing. Collectively, we expect they will provide important insight into potential mechanisms by which a sedentary lifestyle contributes to the increased incidence of cardiovascular disease occurring in individual who are unable to exercise on a regular basis.

## Data Availability Statement

The data that support the findings of this study are available from the corresponding author upon reasonable request.

## Ethics Statement

The study was conducted according to the guidelines for animal care issued by the National Institutes of Health and in accordance with an animal protocol approved by the Institutional Animals Care and Use Committed at Wayne State University. (Animal Welfare Assurance Number A3310-1).

## Author Contributions

BF-K and PM: conceptualization, software, formal analysis, investigation, data curation, writing – original draft preparation, writing – review and editing, visualization, and project administration. BF-K: methodology and validation. PM: resources, supervision, and funding acquisition. All authors contributed to the article and approved the submitted version.

## Funding

This research was funded by National Institutes of Health (R01 HL096787-08). The content is solely the responsibility of the authors and does not necessarily represent the official views of the National Institutes of Health.

## Conflict of Interest

The authors declare that the research was conducted in the absence of any commercial or financial relationships that could be construed as a potential conflict of interest.

## Publisher’s Note

All claims expressed in this article are solely those of the authors and do not necessarily represent those of their affiliated organizations, or those of the publisher, the editors and the reviewers. Any product that may be evaluated in this article, or claim that may be made by its manufacturer, is not guaranteed or endorsed by the publisher.
