# Supplementary material for: Sedentary Conditions Promote Subregionally Specific Changes in Brain-Derived Neurotrophic Factor in the Rostral Ventrolateral Medulla
Source: Front Physiol. 2021 Oct 13;12:756542. doi: 10.3389/fphys.2021.756542 (PMC8548431; doi:10.3389/fphys.2021.756542)
Supplement: Supplementary file 1 [file Data_Sheet_1.PDF]

frontiers

Supplementary Material

Supplementary Table S1. *Post hoc* results of mBDNF and mBDNF/proBDNF ratio within sedentary and physically active groups following a significant interaction between experimental groups and rostrocaudal levels (**for reference see Results and Figures 3D and 3E respectively**).

**Rostrocaudal level comparison**	**mBDNF**		**mBDNF/proBDNF**	
	Sedentary	Active	Sedentary	Active
FN-480 vs. FN-240	0.222	0.166	0.063	0.642
FN-480 vs. FN+240	0.128	0.098	<0.001##	0.507
FN-480 vs. FN+480	0.001##	0.010##	<0.001##	<0.001##
FN-240 vs. FN+240	0.754	0.775	0.024##	0.841
FN-240 vs. FN+480	0.015	<0.001##	<0.001##	0.001##
FN+240 vs. FN+480	0.030	<0.001##	0.016##	0.001##

##, *p* values deemed significance after Holm-Sidak adjustment for multiple comparisons.

Supplementary Table S2. Results from simple main effect testing (Holm-Sidak method) following significant main effect of rostrocaudal distribution of Fl-TrkB and Fl-TrkB/T-TrkB ratio (**for reference see Results and Figures 4B and 4D respectively**).

**Rostrocaudal level comparison**	**Fl-TrkB**	**Fl-TrkB/T-TrkB**
FN-480 vs. FN-240	0.060	0.043
FN-480 vs. FN+240	0.163	0.115
FN-480 vs. FN+480	0.002##	<0.001##
FN-240 vs. FN+240	0.602	0.628
FN-240 vs. FN+480	0.157	0.043
FN+240 vs. FN+480	0.057	0.014

##, *p* value deemed significance after Holm-Sidak adjustment for multiple comparisons.

Supplementary Table S3. *Post hoc* results of nonGlyco-p75 within sedentary and physically active groups following a significant interaction between experimental groups and rostrocaudal levels (**for reference see Results and Figure 5C**) and simple main effect results following a significant main effect of rostrocaudal distribution of Glyco-p75/nonGlyco-p75 ratio (**see Results and Figure 5D**).

**Rostrocaudal level comparison**	**nonGlyco-p75**		**Glyco-p75/nonGlyco-p75**
	Sedentary	Active	Simple main effects
FN-480 vs. FN-240	0.066	0.106	0.040
FN-480 vs. FN+240	0.026	0.006##	0.009##
FN-480 vs. FN+480	<0.001##	0.135	<0.001##
FN-240 vs. FN+240	0.663	0.214	0.513
FN-240 vs. FN+480	0.004##	0.896	0.020
FN+240 vs. FN+480	0.012##	0.171	0.084

##, *p* values deemed significance after Holm-Sidak adjustment for multiple comparisons.

Supplementary Table S4. Simple linear regression results of correlations between total running distance and protein expression levels in the RVLM and $RVLM_{RE}$ of physically active rats (**see Results for reference**).

**Protein**	**RVLM**				**$RVLM_{RE}$**			
	FN-480		FN-240		FN+240		FN+480	
	R^2	*p* value	R^2	*p* value	R^2	*p* value	R^2	*p* value
**proBDNF**	0.168	0.419	0.002	0.933	0.065	0.627	0.002	0.936
**mBDNF**	0.004	0.906	0.051	0.666	0.122	0.498	0.151	0.447
**mBDNF/proBDNF**	0.280	0.281	0.047	0.681	0.036	0.719	0.131	0.481

Abbreviations: BDNF, brain derived neurotrophic factor; mBDNF, mature form of BDNF; proBDNF, pro-form of BDNF; RVLM, rostral ventrolateral medulla; $RVLM_{RE}$, rostral extension of RVLM; FN, facial nucleus.

**Protein**	**RVLM**				**$RVLM_{RE}$**			
	FN-480		FN-240		FN+240		FN+480	
	R^2	*p* value	R^2	*p* value	R^2	*p* value	R^2	*p* value
**Fl-TrkB**	0.312	0.250	0.720	**0.033***	0.031	0.739	0.024	0.769
**T-TrkB**	0.037	0.716	0.157	0.437	0.003	0.916	0.069	0.614
**Fl-TrkB/T-TrkB**	0.220	0.348	0.833	**0.011***	0.078	0.592	0.004	0.904

*, designate significant positive correlation. **Abbreviations:** TrkB, Tropomyosin kinase B receptors; Fl-TrkB, full-length TrkB isoform; T-TrkB, truncated TrkB isoform.

**Protein**	**RVLM**				**$RVLM_{RE}$**			
	FN-480		FN-240		FN+240		FN+480	
	R^2	*p* value	R^2	*p* value	R^2	*p* value	R^2	*p* value
**Glyco-p75**	0.009	0.856	0.100	0.541	0.050	0.670	0.253	0.310
**nonGlyco-p75**	0.015	0.816	0.383	0.190	0.028	0.752	0.076	0.597
**Glyco-p75/nonGlyco-p75**	0.053	0.660	0.192	0.385	0.021	0.785	0.001	0.944

Abbreviations: p75, 75 kDa neurotrophin receptors; Glyco-p75, glycosylated form of p75; nonGlyco-p75, non-glycosylated form of p75.
